# Supplementary material for: Hepatitis E Virus in the Role of an Emerging Food-Borne Pathogen
Source: Microorganisms. 2025 Apr 12;13(4):885. doi: 10.3390/microorganisms13040885 (PMC12029509; doi:10.3390/microorganisms13040885)
Supplement: Supplementary file 1 [file microorganisms-13-00885-s001.zip › microorganisms-3551914-supplementary.pdf]

Table S1: Summary of information about foodborne transmission of HEV.

Observed categories	Animals	Genotype	Confirmed human cases Yes/No
Food-producing animals	Swine	HEV-3, HEV-4 [45,46,47,48,49,50,51,52,53,55,56,58,59,60,61,68,69,70,71,72,73]	No
	Wild Boar	HEV-3 [39,81,82,90,91,92,94]	No
	Deers	HEV-3 [36,81,82,88,93,94,95]	No
	Goat	HEV-3 [83,84]	No
	Sheep	HEV-3 [78,85,86]	No
	Rabbit	HEV-3ra [96,97,98,99,100,101,102]	No
Meat and offal	Swine	HEV-3, HEV-4 [12,60,61,81,111,112,116,117,118,119,120,121,122,123,124,125,126,127,128]	Yes [40,42]
	Cow	HEV-3 [130,131]	No
	Sheep	HEV-3 [86,132]	No
	Wild Boar	HEV-3 [65,70,81,94,133,134,135,136]	Yes [39,34]
	Deers	HEV-3 [70,81,94,133,137]	Yes [36]
	Rabbit	HEV-3ra [98,99,100]	No
Meat products	Swine	HEV-3 [120,128,138,139,140,142,143,144,146,147,150,151,152,153]	Yes [33,41,43]
	Wild boar	HEV-3 [141,145]	No
Milk	Cow	HEV-1, HEV-3, HEV-4 [77,156,157]	No
	Sheep	HEV-1, HEV-3 [13,156,159,160]	No
	Goat	HEV-1, HEV-3, HEV-4 [158]	No
	Camel	HEV-7 [5]	Yes [5]
Shellfish	Mussel	HEV-3 [168,169,170]	No
	Oyster	HEV-3 [170]	No
